# Could Mathematics be the Key to Unlocking the Mysteries of Multiple Sclerosis?

**DOI:** 10.1007/s11538-023-01181-0

**Published:** 2023-06-29

**Authors:** Georgia Weatherley, Robyn P. Araujo, Samantha J. Dando, Adrianne L. Jenner

**Affiliations:** 1 School of Mathematical Sciences, Queensland University of Technology, Brisbane, Australia; 2 School of Biomedical Sciences, Centre for Immunology and Infection Control, Faculty of Health, Queensland University of Technology, Brisbane, Australia

**Keywords:** Multiple sclerosis, Mathematical modelling

## Abstract

Multiple sclerosis (MS) is an autoimmune, neurodegenerative disease that is driven by immune system-mediated demyelination of nerve axons. While diseases such as cancer, HIV, malaria and even COVID have realised notable benefits from the attention of the mathematical community, MS has received significantly less attention despite the increasing disease incidence rates, lack of curative treatment, and long-term impact on patient well-being. In this review, we highlight existing, MS-specific mathematical research and discuss the outstanding challenges and open problems that remain for mathematicians. We focus on how both non-spatial and spatial deterministic models have been used to successfully further our understanding of T cell responses and treatment in MS. We also review how agent-based models and other stochastic modelling techniques have begun to shed light on the highly stochastic and oscillatory nature of this disease. Reviewing the current mathematical work in MS, alongside the biology specific to MS immunology, it is clear that mathematical research dedicated to understanding immunotherapies in cancer or the immune responses to viral infections could be readily translatable to MS and might hold the key to unlocking some of its mysteries.

## Introduction

**Fig. 1:**
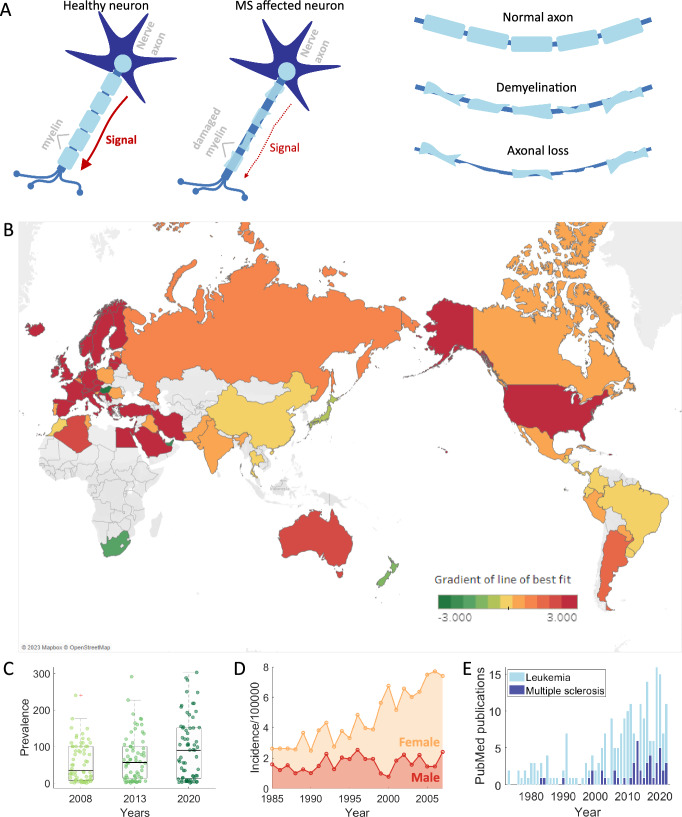
MS disease, prevalence, incidence, and mathematical publication interest. **a** Myelin coating of neurons is degraded in MS patients resulting in demyelination and eventual axonal loss. Destruction of myelin disrupts the ability for signals to transmit along nerve axons. **b** Downloading prevalence data from the MS Atlas (atlasofms.org) and considering only countries with recorded values of prevalence for 2008, 2013 and 2020, the gradient of a line of best fit has been used to colour countries by the rate of change in MS prevalence. The prevalence of MS is the number of individuals with the disease divided by the total population. Note that countries may have obtained values > 3 and < -3 and any countries missing either have no data or data for only one or two years. Most countries have an increasing disease prevalence (orange to red). **c** Box plots demonstrating the increasing prevalence of MS over time. Data were compared using a paired sample *t*-test with a 5% significance and all were found to be significant. **d** Incidence of MS in South East Wales over 22 years plotted by gender (Hirst et al. 2009). Incidence in females is increasing compared to males. **e** PubMed query comparison for mathematical models of leukaemia to models of multiples sclerosis. PubMed query for “Mathematical model” AND (“leukemia” OR “leukaemia”) in light blue. PubMed query for “Mathematical model” AND “multiple sclerosis” in dark blue (Color figure online)

Multiple sclerosis (MS) is a disease of the central nervous system (CNS), brain, retina, and spinal cord, in which the immune system attacks protective coating around nerve axons known as myelin (Dendrou et al. 2015; Hemmer et al. 2015) **(**Fig. 1a). Destruction of the myelin, also referred to as demyelination, exposes nerve axons and disrupts the ability for signals to travel along these axons causing sensory and visual impairments, loss of motor skills and cognitive deficits (Dendrou et al. 2015). Worldwide, more than 2.8 million people have MS and most patients will develop substantial disability during the course of their diseases (Hemmer et al. 2015). MS prevalence and incidence is increasing globally with a significant increase in the proportion of women diagnosed (Fig. 1b–d). Unfortunately, there is still no curative treatment for MS, and the treatments that do exist are not always effective.

For some time now, mathematical descriptions of diseases have been used to improve our understanding of disease origins and potential treatment avenues. Of particular note and relevance are mathematical models in the fields of oncology (Victori and Buffa 2019; West et al. 2022; Bull and Byrne 2022; Engeland et al. 2022; Craig et al. 2020; Altrock et al. 2015; Byrne 2010; Araujo and McElwain 2004), and virology (Smith 2018; Goyal et al. 2019; Perelson 2002; Handel et al. 2018) which have recently started to focus heavily on the immunological aspects of the associated diseases. Despite the multitude of mathematical research capturing the immune response to diseases, there is a significant lack of translation of this research into understanding autoimmune disease such as MS. According to a PubMed query (from 2023), there have been 47 papers published describing mathematical models of MS (Fig. 1e). Over 2.8 million people worldwide have MS (Walton et al. 2020), which is comparative with leukemia, which has over 2.43 million cases (Wang et al. 2022). Comparing the mathematical modelling publication rate for these diseases we see some disparity between the volume of publications. This could be attributed to many factors, for example differences in fatality rate or our understanding of the disease itself. Through rigorously scouring the literature, we classified the modelling of MS into 38 articles that model some aspect of the MS disease using mathematics (Table 1), and an additional 17 that we classified as ‘other’ mathematical or statistical attempts to assist in MS understanding (Table 2). This gave a total of 55 articles, slightly more than was found by the PubMed query. This culmination of work is underwhelming considering the disease impact. Despite this, the collection of mathematical modelling work into MS to date, has made significant impact and illustrates that mathematical models of this disease are both possible and incredibly insightful.

**Table 1:** Mathematical modelling work investigating MS

Reference	Year	Model	Data	Scale	Overview
Balestrino (2009)	2009	ODEs	–	Systemic	System of ODEs was used to test the likelihood of point mutation in gene PTPRC as a cause for cytokine imbalances
Broome and Coleman (2011)	2011	ODEs	–	Cellular	Presented a Biochemical Systems Theory model using ODEs to test drug action in MS patients
Pertsovskay et al. (2013)	2013	ODE	in vitro	Systemic	A kinetic model identified the role of signalling feedback in relation to IFN-$\upbeta$ treatment for MS
Zhang et al. (2014)	2014	ODE	–	Cellular	Dynamical systems theory for a model of Tregs proved recurrent MS arises from a Hopf bifurcation
Kotelnikova et al. (2017)	2017	ODE	EDSS	CNS, systemic	ODE model for axon myelination matched to patient EDSS scores was able to explain disease heterogeneity
Kanna et al. (2017)	2017	ODE	–	Systemic	A model for anti- and pro-inflammatory components was able to predict all subtypes of MS
Montolío et al. (2019)	2019	ODEs	Clinical data	CNS	ODE model relating retinal thickness to EDSS suggested thinning occurs before the appearance of symptoms
Elettreby and Ahmed (2020)	2020	ODE	–	CNS	Stability analysis of a system identified necessary conditions for MS recurrence
Zhang and Yu (2021)	2021	ODEs	–	Cellular	Showed the presence of limit cycles in a simplified model of Teff-Treg interactions
Frascoli et al. (2022)	2022	ODE	Clinical data	Population	A model of population response to DMT fit to clinical relapse measurements, captured the effect of uncertainty
Akaishi et al. (2018)	2018	Logistic map	–	Systemic	A logistic map chaos model was used to explain the disseminated patterns in time and space of MS
Khonsari and Calvez (2007)	2007	PDE	Clinical data	CNS	Proposed a chemotactic model to capture homogeneous concentric demyelination in Baló’s sclerosis
Calvez and Khonsari (2008)	2008	PDE	–	CNS	Adding macrophage recruitment to (Khonsari and Calvez 2007) provided a link between disease aggressivity and spatial patterns
Lombardo et al. (2017a)	2017	PDE	–	CNS	Generalising the models in Khonsari and Calvez (2007), Calvez and Khonsari (2008), a Turing instability was found for chemotactic coefficient values
Bilotta et al. (2019)	2018	PDE	–	Cellular	Classified bifurcation of radially symmetric version of Lombardo et al. (2017b) for the formation of concentric patterns
Bilotta et al. (2018)	2018	PDE	–	Cellular	Investigated the stability of the model in Lombardo et al. (2017b) through a zigzag and Eckhaus instability
Koch et al. (2020)	2019	PDE	MRIs	Systemic, CNS	Proposed a PDE model to learn capillary leakage from MRI data
Hu et al. (2020)	2020	PDE	–	Cellular	Proved the boundedness and global existence of solutions to the chemotaxis model (Lombardo et al. 2017b) and study its stability
Desvillettes et al. (2021)	2010	PDE	–	Cellular	Stability analysis of a reaction–diffusion system showed the appearance of Turing patterns
Desvillettes and Giunta (2021)	2020	PDE	–	Cellular	In the one-dimensional case of Lombardo et al. (2017b), weak solutions were obtained for initial data
Moise and Friedman (2021)	2021	PDE	Clinical data	CNS	System of PDEs described MS plaque growth and exploring treatment combinations
de Paula et al. (2023)	2023	PDE	–	CNS	Studies the influence of innate and adaptive immune responses using a two compartment PDE model
Vélez de Mendizábal et al. (2011)	2011	SDE	CELs	Cellular, Systemic	Using a system of SDEs, concluded that relapsing dynamics derive from Teff-Treg interactions
Bordi et al. (2013)	2013	SDE	Clinical data	Systemic	An SDE model explained the occurrences of relapses and remissions and time spent in either state
Martinez-Pasamar et al. (2013)	2013	SDE	in vitro	Cellular	Fitting the SDE in Vélez de Mendizábal et al. (2011) to flow cytometry for Teff and Treg provided insight into oscillatory dynamics of MS
Pernice et al. (2020)	2020	SSA	Clinical data	CNS, systemic	Lymph node, blood vessels and CNS were simulated using a SSA to reproduce RRMS characteristics
Mohan et al. (2008)	2008	Random graph	–	CNS	A 2D spatial representation of MS disease spread was built using a randomly generated graph
Thamattoor Raman (2012)	2012	Random graph	–	CNS	Extending the model in Mohan et al. (2008), a random graph network was able to capture lesion growth and arrest scenarios
Pernice et al. (2018)	2018	Petri net		Systemic	Description of a Petri Net model to understand Daclizumab treatment of MS
Pernice et al. (2019a)	2019	Petri net		Systemic	Applied a Latin hypercube sampling to Pernice et al. (2018) to reproduce real behaviours of healthy and MS subjects
Pernice et al. (2019b)	2019	Petri net	–	Systemic	Used model by Pernice et al. (2018) to characterise the effects of Daclizumab treatment on RRMS patients
Pennisi et al. (2013)	2013	ABM	–	Cellular, systemic	ABM of the dynamics of RRMS and Teff-Treg cross balancing in genetically predisposed individuals
Pappalardo et al. (2014)	2014	ABM	–	Cellular	Extending the model in Pennisi et al. (2015), they investigated the positive effects of Vitamin D in MS patients
Pennisi et al. (2015)	2015	ABM	–	Cellular, CNS	Extended the model in Pappalardo et al. (2014) to capture the BBB and suggesting new strategies for treatments in MS patients
Pappalardo et al. (2014)	2020	ABM	–	Cellular, systemic	Used the computational model called UISS-MS treatment with DMDs
Pennisi et al. (2020)	2020	ABM	–	Systemic	The model in Pennisi et al. (2015) is used to provide insight into the effects of daclizumab for MS patients
Russo et al. (2022)	2021	ABM	–	Cellular, systemic	Used the computational model UISS-MS to predict relapsing
Russo and Italia (2021)	2021	ABM	–	Cellular	Sensitivity analysis of the UISS-MS (Pappalardo et al. 2014) to understand importance of oligodendrocytes and vitamin D
Sips et al. (2022)	2022	ABM	Clinical data	Population	An in silico clinical trial platform known as MS TreatSim is developed to simulate RRMS patients

The references have been classified by which scale was modelled when considering the following scales: cellular, CNS, systemic, and population. Despite our best efforts, we acknowledge that this exhaustive list may not capture all existing works

**Table 2:** Other relevant MS papers with mathematical or data science techniques

References	Year	Model	Data	Scale	Overview
Crigger (1996)	1996	Causal inference	Clinical data	Population	Adaptation to the uncertainty of MS in women was examined with a causal model
Goodin (2016)	2009	Causal inference	–	Population	A causal scheme to explain MS pathogenesis considering genetic and environmental factors
Sepasian et al. (2014)	2014	Bayesian model	MRI	CNS	Bayesian framework measured lesion change in MRI and detected resolving lesions
Bejarano et al. (2011)	2011	Computational classifiers	MRI	CNS	Constructed computational classifies (Bayesian, neural networks) to find correlates of clinical end points
Meier and Guttmann (2006)	2006	Deterministic	MRI	CNS	Deterministic model for lesion formation and decline over time matched to 997 MRI examinations
Stepanov et al. (2012)	2012	Deterministic	Clinical data	Population	A mathematical model to correctly classify and identify impairment of learning in MS patients
Esteban et al. (2007)	2007	Fractal dimension	MRI	CNS	Assessed the usefulness of Fractal dimension in the measurement of white matter abnormalities
Roura et al. (2021)	2021	Fractal dimension	MRI	CNS	Fractal dimension was demonstrated to identify patients at risk of increased disability over 5 years
Karaca et al. (2015)	2014	Linear model	Clinical data	Population	Linear model fit to patient lesion location and EDSS score to diagnose MS features
Tommasin et al. (2018)	2018	Linear model	Clinical data	Population	EDSS and structural/functional relationships captured with linear model
Bernardo-Faura et al. ( 2021)	2021	Logical model	EAE, clinical data	Systemic	Network model used to uncover features of drug-signalling networks in MS patients
Gulati et al. (2015)	2015	Mixed-effects	CELs	Population	Effect of IFN $\upbeta$-$1\mathrm{b}$ evaluated in patients with RRMS using a mixed-effects model
Velez de Mendizabal et al. (2013)	2013	Mixed-effects	Clinical data, CELs	Population	Non-linear mixed effects model was used to analyse CEL progression and capture patient variability
Kohanpour et al. (2019)	2020	Robust Fuzzy Sliding	–	Systemic	A fuzzy sliding model controlled was designed for the state space model of MS in Kannan et al. (2017)
Goodin (2016)	2016	Probabilistic model	–	Population	Determines the likelihood of developing MS from genetic predisposition vs environmental events
Bielekova et al. (2005)	2005	Stratification algorithm	MRI	CNS	MRI-based stratification algorithm separates patients into clinically meaningful subgroups
Bol et al. (2010)	2010	Structural equation	EDSS	Population	A structural cognitive-behavioural model explained fatigue and physical disability in MS patients
Hu et al. (2022)	2020	Structured functional PCA	MRI	CNS	Proposed a method for capturing temporal and spatial characteristics of lesion in MRI sequences
Krieger et al. (2016)	2016	Topographical model	–	CNS	Model for real-time depiction of disease course and prognosis from clinical/MRI data

The references have been classified according to which scale was modelled when considering four main scales: cellular, CNS, systemic, and population. This represents to the best of our knowledge an exhaustive list, although there may be articles that have been missed despite our efforts

Villoslada and Baranzini (2012) published a “call-to-arms” for the data-science community detailing the opportunities for systems biology to have a major impact on the discovery of biomarkers for a better understanding and diagnosis of MS. More recently in Coggan et al. (2015), discussed the potential for computer modelling in furthering our understanding of demyelinating diseases. Following this, in Pappalardo et al. (2018) published a short review of the efforts from the computational modelling world to understand MS from the context of genetics. Building upon this, the current review aims to serve as a “call-to-arms” for the mathematical modelling community to the open problems for mathematicians in MS.

In this review, we discuss the current success of mathematical research into MS by considering four fundamental mathematical model types: deterministic spatially homogeneous, deterministic spatially inhomogeneous, stochastic spatially homogeneous and stochastic spatially inhomogeneous. We also discuss some of the ingenious ways in which mathematical and statistical methods have been applied to MS data. We broadly classify these works into four disease scales: population, systemic, CNS and cellular; and highlight the work that has been done at each level. We end by presenting the areas which remain open for mathematicians.

## Brief Overview of MS Biology

The immunology of MS is complex and there are still many open questions about the immune pathways involved in the onset and progression of this disease. MS pathophysiology is characterised by the formation of lesions in the CNS. These lesions are caused by immune cell infiltration across the blood–brain barrier (BBB) that promotes neuroinflammation, demyelination (destruction of myelin sheaths), and neuroaxonal degeneration, leading to disruption of neuronal signalling and brain volume changes (Dendrou et al. 2015). We briefly describe below the general immunology of MS relevant to that which has been modelled by the mathematical community. We point readers to other review articles of MS immunology for more in-depth descriptions (Dendrou et al. 2015; Hemmer et al. 2015; Attfield et al. 2022; Arneth 2019; Sospedra and Martin 2005; Høglund 2014; Sellner and Rommer 2020; Lazibat et al. 2018; Li et al. 2018; Grigoriadis and Pesch 2015).

### Setting the Stage of MS Disease

**Fig. 2:**
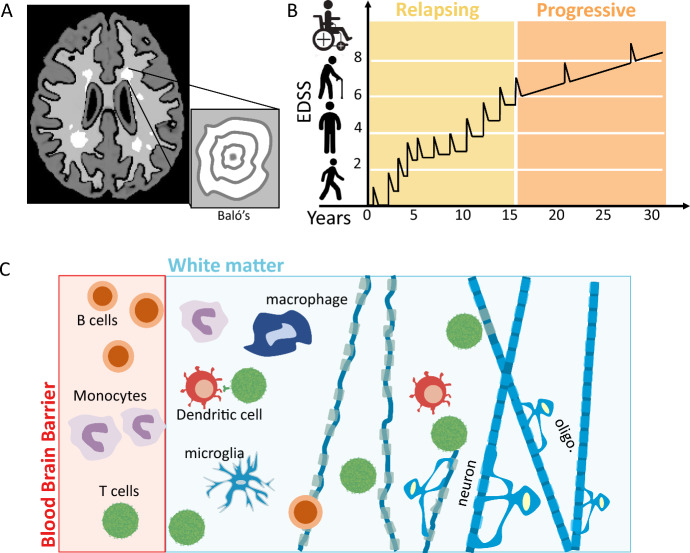
The immunopathology of MS. **a** MRI scans are regularly used to identify lesions, known as contrast enhancing lesions (CELs), seen here as the white areas in the MRI. Most patients have lesions appearing like those shown in the full MRI, however, a rare form of MS known as Baló’s concentric sclerosis shows lesions as concentric rings of demyelinated areas. **b** The main marker of disease progression from relapsing to progressive MS is the expanded disability status score (EDSS). This score ranges from 0 to 10 and represents the level of disability an individual patient experiences at a particular point in time. Over the course of their disease patients undergo relapses in their EDSS score followed by a prolonged period of progression. These relapses correlate to periods of heightened inflammation in the CNS. **c** The immunology of MS is extremely complex and, in this review, we seek to only provide a general overview so as to discuss the mathematical modelling work that has been done. The main cells considered to reside near or within a lesion are T cells, monocytes, B cells, microglial cells, macrophages, and oligodendrocytes (oligo.). T cells, B cells, dendritic cells and monocytes cross the Blood–Brain Barrier (BBB) and infiltrate the white matter of the brain. Dendritic cells present CNS antigens to T cells, which induces their differentiation into effector T cells. Effector T cells, B cells and monocytes promote inflammation which results in the demyelination of neurons. Oligodendrocytes attempt to form myelin in these demyelinated areas and protect demyelinated axons from damage (Color figure online)

MS is a chronic inflammatory disease affecting the brain and spinal cord. MS pathology is characterised by confluent demyelinated areas of the brain and spinal cord that are called plaques or lesions and indicate a loss of myelin sheath and oligodendrocytes (a CNS resident cell whose role is to generate myelin, which is an extension of its membrane) (Dendrou et al. 2015; Kuhn et al. 2019). These lesions can be measured by magnetic resonance imaging (MRI), making MRIs the primary tool in the diagnosis and treatment of MS (Fig. 2a). Active lesions are most frequently localized in the white matter of the brain, but can also be found in the grey matter (Attfield et al. 2022).

There are three main stages of MS (Fig. 2b): relapsing–remitting (RRMS), primary progressive (PPMS) and secondary progressive (SPMS). A relapse in MS is a reflection of an acute focal inflammatory event in the CNS that disrupts neural conduction by damaging myelinated axons leading to lesions (Vélez de Mendizábal et al. 2011). Clinical relapses generally last for a month and can be as frequent as one per year. Within the first few years after diagnosis, most patients experience fluctuations in clinical presentation or relapses and are considered to be in the RRMS stage (Attfield et al. 2022). As time goes on, the relentless and persistent cumulation of severe neurological deficits then dominate the remainder of the patient’s lifetime and patients are either classified as being in the PPMS or the SPMS stage (Dendrou et al. 2015; Attfield et al. 2022). MS disease presentation is extremely heterogeneous across the population, with significant patient-to-patient variations in clinical manifestations and the speed at which patients move from one stage to another (Dendrou et al. 2015). Largely, this is thought to be correlated with the spatiotemporal dissemination of the demyelination within the CNS, which varies from patient to patient (Dendrou et al. 2015).

### Causes and Disease Onset

While the exact MS trigger is still debated, there is substantial evidence that an antigen-specific immune response generated against proteins of the CNS, particularly towards the myelin sheath, is what leads to the destruction of myelin and heightened inflammation eventuating in disease diagnosis (Dendrou et al. 2015). For some time, researchers have put forward the hypothesis that viruses play a role in MS pathogenesis (Oskari Virtanen and Jacobson 2012; Gilden 2005). Early studies suggested the potential role of measles virus in MS (Vandvik et al. 1976; Adams and Imagawa 1962) as well as Epstein-Barr virus (Haahr and Höllsberg 2006). In the last two years, new findings linking MS incidence with EBV infection have strengthened the belief that EBV is a precursor to MS development (Attfield et al. 2022; Bjornevik et al. 2022; Bordon 2022; Sollid 2022; Yates 2022), although the discussion around MS disease onset is still ongoing. For example, recent findings by Ma et al*.* (2023) found that epsilon toxin-producing strains of *Clostridium perfringens* in the gut are able to disrupt the BBB in mice and contribute to inflammatory demyelination, suggesting a role for this bacteria in MS. The combination of viral infection, genetic susceptibility and exposure to environmental factors is believed to lead to the eventual onset of MS. The most common environmental factors linked to MS are Vitamin D deficiency and elevated estrogen levels (Spanier et al. 2015; Hayes and Spanier 2017; Ramien et al. 2016).

### Immunopathology of MS

Results from immunological, genetic and histopathological studies of MS patients have shown that the immune system plays a key role in disease initiation and progression (Hemmer et al. 2015). T and B lymphocytes have long been considered the major players in MS immunopathology (Sellner and Rommer 2020; Li et al. 2018). Very early in the disease, patients with RRMS show widespread inflammatory infiltrates, with most evidence to date identifying populations of CD4 + T cells, CD8 + T cells, B cells and monocytes within lesions (Fig. 2c). The subsequent destruction of myelin-producing oligodendrocytes by these cells leads to the formation of acute lesions in early disease stages.

In this review, we consider T cells can be broadly grouped into effector or regulatory cell types based on their mechanism of action. Regulatory T cells (Tregs) suppress disease development through the inhibition of effector T cells (Teffs) (Bar-Or and Darlington 2011). In contrast, Teffs are thought to play a role in heightening inflammation at the lesion site and increasing myelin degradation and oligodendrocyte death. Both CD4 + and CD8 + T cells can exhibit effector and regulatory activities and while MS was long believed to be a CD4 + T cell disease (Kaskow and Baecher-Allan 2018), CD8 + T cells have been shown to dominate the T cell infiltrates in active MS lesions (Attfield et al. 2022). B cells and Myeloid cells (macrophages, dendritic cells and microglial) control T cell activation through antigen-presentation (Filippi et al. 2018). The binding of antigen to the cell surface activates dendritic cells which communicate with naïve CD4 + T cells and shape the adaptive immune response (Grigoriadis and Pesch 2015). Monocytes and macrophages are found in high numbers in the CNS of MS patients and thought to activate or control T cell activity at the lesion site (Bar-Or and Darlington 2011). Two key CNS-resident cells in MS disease are oligodendrocytes and microglial. Oligodendrocyte cells are crucial for myelin repair. Microglial are the tissue-resident macrophages of the CNS, and although their role in MS pathogenesis is inconclusive it is thought to be in an activation or stimulation role (Attfield et al. 2022).

### Clinical Measurements of MS Disease

**Fig. 3:**
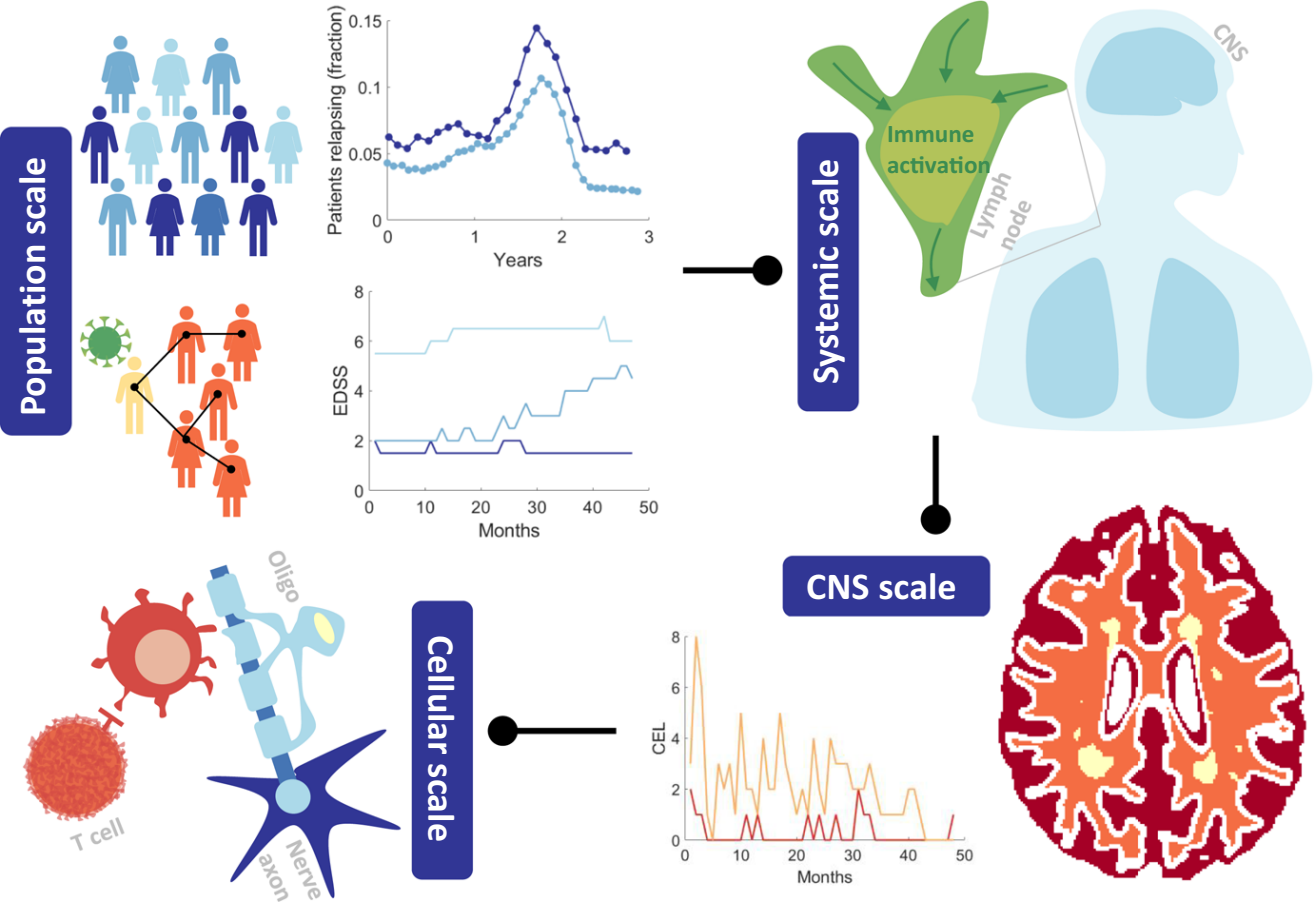
MS: a multiscale mathematical modelling problem. MS occurs across multiple scales which we classify generally as: *population, systemic, CNS* and *cellular*. Population scale considers modelling efforts aimed at understanding the environmental and genetic risk factors associated with developing MS as well as cohort relapses under drug treatments such as IFN-$\upbeta$ (dark blue) and Fingolimod (light blue) (Frascoli et al. 2022; Roos et al. 2020). Population modelling efforts can benefit from the inclusion of clinical data such as Expanded Disability Status Scale (EDSS) records (Vélez de Mendizábal et al. 2011; Bagnato et al. 2003). Systemic level modelling takes into account how the lymph nodes and other areas of the body play a role in MS disease progression and treatment. When considering the CNS (brain and spinal cord), MRI measurements can be used to determine location and intensity of lesions and obtain a per-patient measurement for the number of Contrast Enhancing Lesions (CELs) (Vélez de Mendizábal et al. 2011; Bagnato et al. 2003). Focusing more on the cellular and molecular interactions gives the cellular scale of modelling. These models may be more concerned with individual cell activity, stimulation, signalling and myelin regeneration. See Tables 1 and 2 for a full list of models classified to these scales (Color figure online)

MRIs are the primary measurement of patient disease, providing visible locations of patient lesions. While most patient’s lesions exhibit no discernible, predetermined pattern, in a rare form of MS known as Baló’s concentric sclerosis (Fig. 2b), demyelination occurs in striking concentric patterns (Khonsari and Calvez 2007). With increasing age and disease duration, new focal inflammatory lesions become less frequent in MS patients, whereas some demyelinated lesions remain chronically active. Often these focal inflammatory lesion events are denoted contrast enhancing lesions (CELs) on T1-weighted images and can be counted and tracked over time (Fig. 3). CELs are also considered markers of BBB breakdown (Bagnato et al. 2003; Campbell et al. 2012). The BBB is an endothelial cell barrier that restricts immune cell trafficking into the CNS; however, in patients with MS the BBB can become damaged, thereby allowing immune cell trafficking into the brain which in turn causes inflammation and subsequent demyelination. Another measure of MS clinical outcomes is brain volume loss, where the rate of brain volume loss correlates with disease severity (Radue et al. 2015; Stefano et al. 2016). An additional important clinical measurement is the expanded disability status score (EDSS), which tracks an individual patient’s disability over time (Fig. 2a).

### Treatment of MS

Current therapeutics available for MS patients are mostly effective for RRMS patients and can readily reduce the frequency of relapses, yet are seemingly unable to perturb the pathological processes associated with disease progression (Attfield et al. 2022). Current management strategies are focused on treating attacks, ameliorating symptoms and reducing biological activity through disease-modifying therapies (DMTs) (Sellner and Rommer 2020; Hauser and Cree 2020). One of the first approved DMTs was interferon-beta (IFN-$\upbeta$) which is known to reduce the frequency of MS relapses (Hauser and Cree 2020). Monoclonal antibodies such as natalizumab, and daclizumab have been developed more recently, and are highly effective in reducing relapses and slowing disease progress in RRMS (Hauser and Cree 2020; Schippling and Martin 2008). Unfortunately, there is no all-round curative treatment, and patients ultimately progress regardless of therapy. Furthermore, treatments are ineffective once an individual has reached the PPMS or SPMS stage.

## The Status of Mathematical Modelling of MS

Despite the small body of mathematical work studying MS (Tables 1 and 2), mathematicians have already provided insight at four main scales of MS: population, systemic, CNS, and cellular (Fig. 3). Investigations that probe questions around population-level disease dynamics, such as susceptibility and response to treatment, can be considered “population scale”. In contrast, mathematical models capturing the systemic intra-patient dynamics, such as lymph node activity and cytokine signalling, are considered to account for the “systemic scale”. Many models have exclusively focused on the activity within the CNS, given that is where MS disease presentation arises, and hence are at the “CNS scale”. Whereas some models have centred around the “cellular scale”, which considers how individual cells interact with myelinated axons and other CNS-resident cells.

The earliest mathematical applications to MS can be found in models using causal inference (Goodin 2009; Ramagopalan et al. 2010; Crigger 1996). Causal inference and causal models are mathematical techniques used to represent causal relationships within an individual system or population. Subsequently, these models have been used to facilitate inferences about causal relationships from MS patient data. Apart from this, the predominant mathematical work in MS includes spatial and non-spatial deterministic systems that capture various aspects of the disease aetiology (Vélez de Mendizábal et al. 2011; Khonsari and Calvez 2007; Frascoli et al. 2022; Martinez-Pasamar et al. 2013; Kotelnikova et al. 2017; Elettreby and Ahmed 2020; Montolío et al. 2019; Broome and Coleman 2011; Koch et al. 2020; Lombardo et al. 2017a; Calvez and Khonsari 2008; Bilotta et al. 2019, 2018; Desvillettes and Giunta 2021; Hu et al. 2020; Desvillettes et al. 2021; Moise and Friedman 2021). Motivated by the inherent stochasticity of this disease, stochastic computational models have also been considered more recently (Pennisi et al. 2015, 2013; Pappalardo et al. 2014, 2018). To summarise the work that has been done at the different disease scales, we categorise and discuss the published mathematical modelling studies according to their overarching mathematical assumptions: non-spatial deterministic, spatial deterministic, non-spatial stochastic and spatial stochastic modelling. Tables 1 and 2 provide an annotated list of all publications, to the best of our knowledge, using mathematics to model some aspect of MS.

### Spatially Homogeneous Deterministic Models of MS

**Fig. 4:**
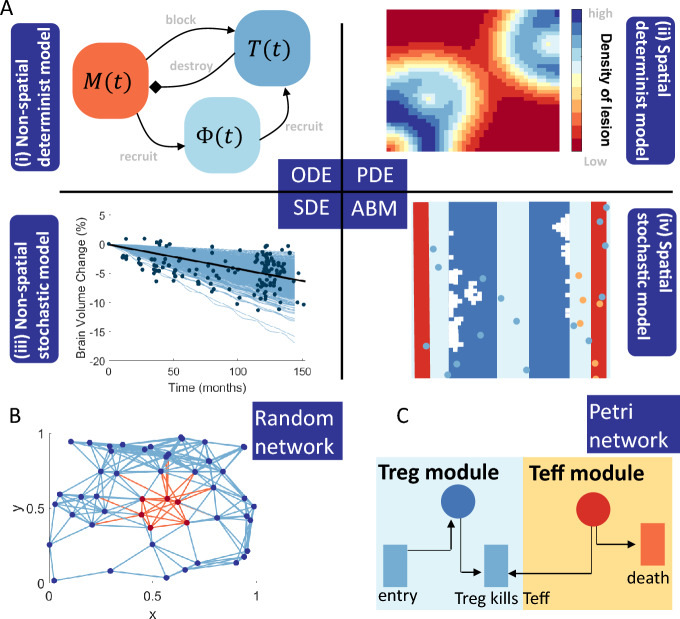
Summary of mathematical modelling techniques that have been applied to MS. **a** Generally, there are four main modelling regimes in mathematical biology: non-spatial deterministic, spatially deterministic, non-spatial stochastic and spatially stochastic regimes. These four regimes usually rely on modelling techniques such as ODEs, PDEs, SDEs and ABMs. (i) ODEs represent a mean-field approximation to population level dynamics, for example destruction of myelin $M(t)$ occurs by T cells $T(t)$ which are recruited by macrophages $\Phi (t)$. (ii) Incorporating the spatial location of these cells provides a density of a particular entity at a position $(x,y)$ and, as such, builds a picture of spatial spread of a lesion in the CNS. (iii) Given that heterogeneity and variability is evident in MS patient data, particularly Brain Volume Change (%) (Stefano et al. 2016), capturing that with a deterministic model is not possible, so we introduce noise, either inherent or explicitly into the ODE model to capture this variability. (iv) Modelling stochasticity in individual cellular interactions and movements in MS gives rise to ABMs. **b** Another technique employed by modellers for MS is random network models, which can be considered a spatially stochastic model (Mohan et al. 2008; Thamattoor Raman 2012). **c** Lastly, another technique is Petri net, which is more of a homogeneous stochastic model (Pernice et al. 2018, 2019a, b) (Color figure online)

Non-spatial (or spatially homogeneous) deterministic mathematical models are regularly used in mathematical modelling of biological phenomenon when a mean-field estimate or a well-mixing assumption for the populations of interest is acceptable. In deterministic models, the focus is on the non-random interactions of the disease, and predicting average population counts over time, as opposed to spatial densities (Fig. 4a). These models usually consist of Ordinary Differential Equations (ODEs) which capture the change in some key aspect of the biology over time. For example, An ODE system can be used to capture the change in total Myelin $M(t)$ and immune cells, such as T cells $T(t)$ or macrophages $\Phi (t)$ over time $t$. ODE models to date have considered the change in total inflammatory T cells (Broome and Coleman 2011; Balestrino 2009; Zhang et al. 2014; Zhang and Yu 2021), IFN signalling molecules (Pertsovskaya et al. 2013) or axon damage (Kotelnikova et al. 2017; Montolío et al. 2019).

One of the earliest instances of an ODE model of MS was implemented by Broome et al*.* (2011), who used biochemical systems theory (BST) to capture cellular pathways resulting in oligodendrocytes death (Fig. 2). BST uses ODEs to capture biochemical processes (Voit 2000) and the model developed by Broome et al*.* consists of 79 independent variables, 77 dependent variables and 77 system equations modelling the intricate aspects of the intracellular death-response network for oligodendrocytes. Their work highlighted the viability of oligodendrocytes as therapeutic targets and this notion has recently received attention experimentally (Chen et al. 2019). Broome et al*.* posited that their method of locating trigger points in the model that lead to diseased states could have future merit in the development of MS treatments, although the large number of unknown variables and parameters could be a challenge for a BST model of MS.

Moving from Broome et al*.*’s model (Broome and Coleman 2011) at the molecular/cellular scale to the CNS scale, to explain the stages of MS disease progression and recurrence, Elettreby and Ahmed (2020) developed a system of three ODEs for healthy brain cells $x(t)$, affected brain cells $y(t)$, and a harmful “effector” such as immune cells $v(t)$:

1 $$\begin{aligned} \frac{dx}{{dt}} & = rx\left( {1 - x} \right) - bxv, \\ \frac{dy}{{dt}} & = bxv - ay, \\ \frac{dv}{{dt}} & = cy - dxv - kv. \\ \end{aligned}$$

Unlike the model by Broome et al*.*, their model was more general in capturing the disease evolution, where $r$ represented the growth rate of healthy brain cells, $b$ the rate healthy cells are attacked, $a$ the rate at which affected brain cells die, and $k,d$ the rate at which the “effector” dies. Conducting a stability analysis, they reproduced conditions under which stable or oscillatory disease dynamics were obtained. The oscillations of the model were found to arise due to a mathematical instability and could be representative of relapses, such as those in RRMS. Their work opens the door for future analysis and extensions whereby the inclusion of explicit immune cell actions could help to understand which biological conditions give rise to these oscillations. In particular, inspiration for extending this model could be taken from the work by Zhang et al*.* (2014), who developed a set of four ODEs capturing specific interactions of CNS immune cells (population $v(t)$ in Eq. (1)): antigen-presenting cells, Tregs, Teff and antigen.

Taking a slightly different approach but still at the CNS scale, Kotelnikova et al*.* (2017) and subsequently Montolío et al*.* (2019), used ODE systems to capture axon volume changes over time. Kotelnikova et al*.* (2017) developed a system of four ODEs that captured immune attack, alongside demyelination, remyelination and axonal loss. Unlike Zhang et al*.* (2014), their model did not explicitly model the action of immune cells and only captured the change in myelinated axons, demyelinated axons, remyelination capacity and axon degeneration. Their model was fit to EDSS data of a clustered, longitudinal cohort of 66 MS patients and then validated using EDSS data and brain volume time series of a second cohort of 120 MS patients. Their work supported the conceptualisation of MS as a single, progressive disease, with dynamic CNS damage driving heterogeneity.

With a similar focus on axon myelination like that by Kotelnikova et al*.* (2017), Montolío et al*.* (2019) developed a system of ODEs to relate retinal nerve fibre layer (RNFL) thickness in MS patients with their EDSS scores. The ODEs captured the evolving proportions of healthy and damaged axons by their RNFL thickness, alongside axonal degeneration. Clinical data from 114 ophthalmologically-evaluated patients was clustered through a k-means clustering algorithm and EDSS scores of each cluster were fit to a probability distribution. This data was used to calibrate the model and then a further 70 patient measurements were used to validate. They found that RNFL thinning was occurring prior to disability presentation. Unfortunately, their model only implicitly captured the immune system, and future work could look to better combine both Kotelnikova et al.’s and Montolío et al.’s models to consider specific cells of the immune system.

Moving now to the population-scale, the recent work of Frascoli et al*.* (2022) developed a population-level model of RRMS patients under relapse supressing therapies. The system of ODEs contained three mutually exclusive compartments of patients in a pre-relapse state, post-relapse state, and currently relapsing state. Their model considered four parameters capturing the duration of post-relapse, the time needed to change from pre-relapse to relapse, and the time needed for a patient to move to a post-relapse status, and the initial number of relapsing patients. Utilisation of existing density curves of MS relapses allowed for model formulation and calibration, with four curves of the following immunotherapies selected: interferon beta-1a S.C. (IFN-$\upbeta$-1a S.C.), interferon beta-1b (IFN-$\upbeta$-1b), natalizumab, and fingolimod. Unlike other models that strove to capture individual patient disease courses such as that by Kotelnikova et al*.* (2017), the model reproduced a patient group response to these treatments. Future work is required in investigating the possible presence of underlying, universal features of treatment switch dynamics.

### Spatial Deterministic Models of MS

When considering the spatial density of cells or myelin in MS (i.e. the CNS scale in Fig. 3), modellers often turn to Partial Differential Equations (PDEs) as a means of determining spatial estimates (Fig. 4). While there have been some PDE driven investigations of MS, most centre around global asymptotic stability analysis of models relating to the rare MS subtype called Baló’s concentric sclerosis (Khonsari and Calvez 2007; Lombardo et al. 2017a; Calvez and Khonsari 2008; Bilotta et al. 2019, 2018; Desvillettes and Giunta 2021; Hu et al. 2020) (Fig. 2). The motivation for spatial modelling of this MS subtype arises from the concentric rings in lesion growth, which are reminiscent of Turing patterns or reaction–diffusion problems. While these studies have provided significant understanding particularly to this subtype of MS, and on how to analyse chemotactic models of spatial phenomenon, they are yet to provide insight into the broader disease dynamics. The remaining PDE models of MS, have considered capillary leakage (Koch et al. 2020) or plaque growth and treatment (Moise and Friedman 2021), and most recently (published this year) the interplay of the innate and adaptive immune responses in the CNS (Paula et al. 2023), highlighting the vast potential for PDE modellers to provide novel spatial insight into MS.

Khonsari and Calvez were the first to attempt to identify the potential cause of concentric lesion phenomenon using chemotactic cellular models of MS (Khonsari and Calvez 2007; Calvez and Khonsari 2008). They developed a PDE model, motivated by Liesegang rings, which arise from a periodic precipitation process involving three chemical species,

$$A + B \to D$$

where $B$ is uniformly distributed and $A$ propagates with a diffusion front. As the reaction proceeds, consecutive bands of precipitate form (Khonsari and Calvez 2007). Connecting Liesengang rings to the MS physical system, Khonsari and Calves proposed that a protective substance, secreted by the attacked oligodendrocytes, diffuses through the domain preventing demyelination by the activated macrophages which undergo chemotaxis towards an attraction signal (Khonsari and Calvez 2007; Calvez and Khonsari 2008). Through their work, they derived the following system which suggests the appearance of rings caused by chemotactic mechanisms:

2 $$\begin{aligned} \frac{\partial m}{{\partial t}} & = \underbrace {{D{\Delta }m + \lambda m\left( {\overline{m} - m} \right)}}_{{{\text{Macrophage }}\,{\text{front}}}} - \underbrace {{\nabla \cdot \left( {\chi m\left( {\overline{m} - m} \right)\nabla c} \right)}}_{{{\text{Macrophage}}\,{\text{ recruitment}}}}, \\ \frac{\partial d}{{\partial t}} & = \underbrace {{F\left( m \right)m\left( {\overline{d} - d} \right)}}_{{{\text{Destruction}}\,{\text{ of}}\,{\text{ myelin}}}}, \\ & \underbrace {{ -\epsilon {\Delta }c + \alpha c = \mu d}}_{{{\text{Production }}\,{\text{and}}\,{\text{ diffusion }}\,{\text{of}}\,{\text{ signal}}}}, \\ \end{aligned}$$

where $m$ is the density of activated macrophages, $c$ is the concentration of attraction signal and $d$ is the density of destroyed oligodendrocytes. Macrophages undergo diffusion, with diffusion coefficient $D$ and are also undergoing logistic growth at a rate $\lambda$ with capacity $\overline{M }$, similar to how Elettreby and Ahmed (2020) modelled healthy brain cell regeneration. Macrophages are also undergoing chemotaxis with bias coefficient $\chi$. Oligodendrocytes are being destroyed at a rate $F(m)$ and capacity $\overline{d }$. Lastly, $\sqrt{\epsilon /\alpha }$ is the approximate range of the signal. While the model can capture Baló’s concentric sclerosis, it does not account for the slow regeneration of myelin by oligodendrocytes typical of other MS subtypes. The model presented differs also to the ODE model by Zhang et al*.* (2014), who chose to omit explicitly modelling macrophages.

Lombardo et al*.* (2017a) generalised Khonsari and Calvez’s model above by choosing a different analytical form for the chemotactic sensitivity function describing macrophage chemotaxis by replacing the quadratic $m\left(\overline{m }-m\right)$ with a hill function $m/(\overline{m }+m).$ In addition, they also explicitly modelled the diffusion, production, and decay of chemokine. Ultimately, they were able to observe consistency between numerical simulations of both plaque shape and size, and MRI data. Importantly, their work differed from Khonsari and Calvez (2007) by supporting concentric ring lesion formation despite the omission of oligodendrocyte-driven cytokine production. The authors recognised that white matter heterogeneity limited their numerical simulations, and that their model overlooked the anti-inflammatory role of cytokines in remyelination. Numerous later studies then subsequently analysed the global existence of solutions and stability of Lombardo et al.’s model (Bilotta et al. 2019, 2018; Desvillettes and Giunta 2021; Hu et al. 2020; Desvillettes et al. 2021).

Distinct from the work by Lombardo et al., Koch et al*.* (2020) proposed a new mathematical model to learn capillary leakage coefficients from dynamic susceptibility contrast MRI data. They developed a perfusion model on a subvoxel scale by including the capillary network structure and derived a transport model for brain tissue perfusion. In this modelling format, blood vessels represented by a network of cylindrical segments are embedded into the extravascular space, represented by a homogenized three-dimensional continuum. They used their model to obtain the contrast agent concentration distribution in a single MRI voxel during a perfusion.

Still within the CNS modelling scale, and motivated by a desire to quantify the implications of treatment timing and combinations of current treatments, Moise and Friedman (2021) developed a mathematical model of MS plaques. This model was the first to explicitly quantify the effect on plaque growth of cytokines, macrophages and T cells as well as drug treatments (IFN-beta, glatiramer acetate, and natalizumab), although they similarly to Khonsari and Calvez (Khonsari and Calvez 2007; Calvez and Khonsari 2008) chose to model oligodendrocyte activity (see Eq. (2)). Their model consisted of 15 PDEs which captured all aspects of the cellular immunology of MS in the CNS. The authors compared their simulations of plaque volume to different sets of clinical data and showed good qualitative agreement. Their additional exploration of drug combinations even gave an incidence of decreased initial plaque volume after 270 days. While the work was novel in being the first mathematical model to account for plaque geometry, their model restricts plaque geometry to a spherical domain, despite it being clear from MRIs that plaques are characterised by heterogeneous growth. In addition, unlike the PDE works of Koch et al*.* (Koch et al. 2020) and Khonsari and Calves (2007) and Calvez and Khonsari (2008), their model was much more complex and detailed in the mechanisms modelled.

### Spatially Homogeneous Stochastic Modelling of MS

Clinical measurements of MS, such as EDSS and CEL measurements, paint a picture of a highly stochastic temporal disease. For this reason, mathematicians have sought to investigate how stochastic (noisy) models might explain inter- and intra-patient variability, whilst considering spatial homogeneity. A range of techniques have been investigated including stochastic differential equations (SDEs, Fig. 4) (Vélez de Mendizábal et al. 2011; Martinez-Pasamar et al. 2013; Bordi et al. 2013), a stochastic simulation algorithm (Pernice et al. 2020), mixed effects models (Gulati et al. 2015; Velez de Mendizabal et al. 2013), stochastic symmetric nets (Fig. 4) (Pernice et al. 2018, 2019a, b), in silico clinical trials (Sips et al. 2022), and statistical models (Hu et al. 2022; Goodin 2016).

To gain insight into the cellular events leading to the relapsing dynamics of MS, Vélez de Mendizábal et al*.* (2013) developed a mathematical model of the Teff and Treg interactions and their effect on healthy myelin. Their model captured these cells at the CNS and systemic scale. Unlike the models by Pernice et al*.* (2018) and Moise and Freidman (2021) who used mass-action or linear reaction terms, the authors chose to model the interactions between Teff and Treg cell populations using hill functions. Their model used a system of 6 ODEs with stochastic inputs to capture the natural stochasticity in immune stimulation. The authors hypothesised that cross-regulation between Teffs and Tregs coupled with stochastic processes (e.g. infections) was able to buffer oscillations in the functioning of the immune system, causing the initiation of an immune response when required. They suggested that irrespective of additional environmental triggers, weakness in the Teff-Treg feedback loop prompts immune-mediated RRMS. The authors acknowledged that alongside constraints imposed by clinical stochasticity and biological unknowns, their work omitted immune response aspects such as innate immune activity, which may be crucial for a comprehensive depiction of MS disease.

With therapeutic implications in mind, Martinez-Pasamer et al*.* (2013) adapted the T cell model of Vélez de Mendizábal et al*.* (2011) to experimentally validate the postulate that relapsing–remitting disease behaviour is driven by lymphocyte cross-regulation. Their version of Vélez de Mendizábal et al*.* (2011)’s model considered only four ODEs, omitting the variables for tissue behaviour and focusing only on the immune cell populations. By analysing Teff, Treg and microglia behaviour obtained from flow cytometry data, their simulations suggested that Treg activation as a key element of autoimmune susceptibility of MS. Assessing the role of B-cell depletion induced by anti-CD20 therapy, they observed that depletion does decrease Teff expansion. However, B cell depletion also significantly effected Tregs which resulted in worsening of the disease. Further model validation using experimental testing was also suggested, involving various T cell viability assays. Petri Nets (PNs) are widely recognised to be a powerful modelling tool for studying biological systems (Marsan et al. 1998; Hardy and Robillard 2004; Koch 2010). These are bipartitie directed graphs with two types of nodes, places (circles) and transitions (boxes). Places correspond to state variables e.g. cell types, and transitions correspond to events, e.g. death (Fig. 4c). A place can contain a number of tokens and a transition is usually enabled if all places connected to it have sufficient tokens. Consider in this context a scenario where Treg and Teff cells are places and transitions correspond to the induction of a state change such as the killing of Teff cells by Treg cells. The system evolution is given by the firing of an enabled transition, where a fixed number of tokens are removed from the input place and added to the output place. In stochastic symmetric petri nets, the firing of each transition is assumed to occur after a random delay from the enabling time.

Pernice et al*.* developed a body of work looking at extensions of PNs (Pernice et al. 2018, 2019a, b, 2020) and implemented a stochastic PN to capture the immune response in RRMS by considering lymph nodes, blood vessels, Teff, Treg, oligodendrocytes and the CNS explicitly. Their modelling of the cellular interactions between Teff, Treg and oligodendrocytes was similar to the work of Moise and Freidman (2021), however, neither groups modelled the intracellular death responses like Broome and Coleman (2011). Their simulation results for the administration of daclizumab confirmed the importance of timely intervention when attempting to favourably alter patient disease course through treatments.

Taking a data science driven approach using matrix decomposition, Hu et al*.* (2022) used individual lesion voxel intensity trajectories to develop a statistical model using structural principal component analysis (PCA) for MS lesion evolution. While others have used a variety of computational methods to extract useful insight from MRIs (Sepasian et al. 2014; Bejarano et al. 2011; Meier and Guttmann 2006). Hu et al*.*’s work was novel in accounting for the multilevel structure of the MRI data when evaluating sample properties of hypothesis tests of the effect on lesions of MS therapies. In addition, this study used the images of 36 patients assessed monthly with either relapsing remitting or secondary progressive MS, ultimately indicating significant statistical differences between lesion evolution of untreated and treated participants. The authors recognised the limitations of their work due to the unpredictable spatial and temporal nature of lesion development creating unbalanced data, for which there is no existing study of hierarchical hypothesis testing.

### Spatially Stochastic Models of MS

As computational power has increased, our ability to capture biological interactions as spatially stochastic events, as opposed to mean-field average rates, has evolved. Agent-based models (ABMs, Fig. 4a) are an exciting computational modelling tool where individual *agents* obey certain rules driven by probabilities. Generally, ABMs in MS consider cells as agents and each cell has a position in either 2 or 3 dimensions (Pennisi et al. 2015, 2020, 2013; Pappalardo et al. 2014, 2018; Russo et al. 2022; Russo and Italia 2021).

The first ABM developed to capture MS disease kinetics was designed by Pennisi et al*.* (2013) to capture the Teff-Treg cross balancing in RRMS in genetically predisposed individuals using Netlogo (an ABM platform). A 51 $\times$ 51 cell grid was initialised in which agents moved and interacted based on a Von Neumann neighbourhood. Their model was later extended by Pennisi et al*.* (2015) and Pappalardo et al*.* (2014) to capture the effects of Vitamin D and different treatment strategies. All the models by Pennisi et al*.* (2015, 2013) and Pappalardo et al*.* (2014), considered variations of the immunological dynamics of MS at the CNS-scale. Simulating unique patients through different initial seeds, Pennisi et al*.* (2015) varied model parameters to implicitly model treatments. From 900 simulations, consisting of 9 scenarios of 100 patients/seeds, the authors were able to suggest that there is greater treatment effectiveness from preventing BBB opening than from attempting recovery of BBB functionality. A minor limitation of this platform is the on-lattice nature of the cellular movement and interaction rules. Future work could consider extensions of this model where the complex architecture of white matter is included using diffusion tensor MRIs.

Further developing the ABM in Pennisi et al. (2015, 2013) and Pappalardo et al. (2014) to a multiscale multiorgan simulator, i.e. a systemic-scale model of MS, Pappalardo et al*.* (2018) sought to predict the evolution of relapsing MS and investigate treatment effects in a greater capacity. Prediction robustness was tested by utilising known predictive factors such as age, vitamin D levels and smoking to simulate poor prognosis. To inform their model, they obtained MRI lesion load and other features of six MS patients with heterogeneous relapsing–remitting disease courses. The authors simulated individual scenarios consistent with each patient’s clinical and MRI history. Noting its capacity to anticipate relapse timing of two patients, the authors recognised the distinctive potential of their model in generating personalised outcomes through specific patient data. They proposed the further development of a model through additional genetic, immunological, and environmental considerations to ultimately predict individual patient disease dynamics and inform therapeutic interventions. Interestingly, no ABM to date has been developed that considers the MS disease at the population level and this could be a useful extension of current models developed for the COVID-19 pandemic, to investigate the implications of EBV spread.

Taking a very different approach to agent-based modelling, Mohan et al*.* (2008) and Thamattoor Raman (2012) presented a unique method for spatially capturing the random spread of disease using an undirected, fixed radius random graph $G(n,r)$ where $n$ were nodes representative of cell bodies (functional units) and edges connecting nodes represented axons (connections between function units). Generating uniformly random placement of nodes in a grid $\left[\mathrm{0,1}\right]\times [\mathrm{0,1}]$ and imposing connections between nodes of Euclidean distance $<r$, damage can be modelled as randomly spreading throughout the network by initialising some damage in the centre and with radial distance $RO{I}_{t=0}$. Unlike the stochastic spatial models above, this model did not explicitly model individual immune cell actions, but instead implicitly captured disease spread through the CNS using a network. Simulating their model, Mohan et al*.* (2008) and Thamattoor Raman (2012) found that the spread of the pathologic process of MS can be arrested by programmed cell death in the periphery of the lesions. This is an interesting finding and relates back to the work of others, such as the BST by Broome and Coleman*.* (2011), around the importance of understanding the role of oligodendrocytes in MS. Similar applications of this modelling technique can be found in the avian influenza epidemic (Kim et al. 2010) and more general studies of tissue damage (Kim et al. 2009).

### Other Examples of Mathematical and Computational Methods in MS

The above review represents a description of more classical modelling techniques in widespread use within the mathematical biology community. There are also a number of studies that have sought to apply less conventional methods to understand MRIs (Sepasian et al. 2014; Karaca et al. 2015; Roura et al. 2021), EDSS data (Bol et al. 2010) and other MS-related clinical measurements (Tommasin et al. 2018; Krieger et al. 2016). For example, fractal dimension has been used by authors to analyse abnormalities in patient MRIs (Roura et al. 2021; Esteban et al. 2007). Roura et al*.* (2021) determined the fractal dimension of MRIs from 146 patients with RRMS. In this context, fractal dimension provides a numerical characterisation of fractal patterns in the brain, and can thereby provide a measure of brain morphology, which can in turn detect CNS damage. In particular, the higher the fractal dimension, the more complex and healthier the brain. From their analysis, these authors concluded that fractal geometry of the brain could identify patients at risk of increasing their disability in the next five years.

The robustness of brain structural networks can be estimated from diffusion MRI data and may extend to patient cognition. Farooq et al*.* (2020) investigated whether measures of network robustness can explain cognitive impairment in MS patients using the Ollivier-Ricci curvature (ORC) (Sia et al. 2019). The notion of curvature, from Riemannian geometry, quantifies how geodesic paths converge or diverge. The ORC captures the notion of network flows of shortest paths via the Wasserstein distance, wherein a negatively curved edge is a “bottleneck” (Sia et al. 2019). The authors assessed whether local or global (whole brain) robustness differs between cognitively impaired and non-impaired patients. Brain structural network robustness and centrality showed significant correlations with cognitive impairment. Measures of network robustness and centrality also identified specific cortical areas relevant to MS-related cognitive impairment.

To assess whether MS subgroup differences are sustained long term, Bielekova et al*.* (2005) used patient MRI markers of inflammation and axonal damage to design a stratification algorithm. Through a cross sectional analysis of 71 untreated MS patients, the algorithm divided MS patients into meaningful long-term groups. By testing the model against a longitudinal cohort of 71 patients, the authors were able to distinguish four subgroups with persistent MRI measured differences over 8 years. The authors acknowledged that the chosen MRI markers were not complete measures of MS processes, and that future work is required in understanding the mechanisms that produce the various disease phenotypes.

## Discussion

Our incomplete understanding of the causes and pathways involved in the onset and progression of MS limits our ability to effectively treat this complex neurological disease (Attfield et al. 2022). Mathematical modelling of MS to date has highlighted the potential impact mathematicians could have in the diagnosis and treatment of this disease, albeit significantly more work is needed. While diseases such as cancer, HIV, malaria and even COVID-19 garner huge attention from mathematical modellers, MS has gone relatively unnoticed. Considering that the common element linking the aforementioned diseases with MS is the immune system, we believe that the time is ripe for mathematicians to embrace the modelling opportunities presented by MS.

To date, we have seen publications using the four main modelling techniques; ODEs (Frascoli et al. 2022), PDEs (Moise and Friedman 2021), SDEs (Vélez de Mendizábal et al. 2011), and ABMs (Pennisi et al. 2015); as well as some less common modelling techniques; fractal dimension (Roura et al. 2021), robust fuzzy sliding door (Kohanpour et al. 2019) and random graphs (Mohan et al. 2008). We’ve also detailed models that use MRI data (Koch et al. 2020), EDSS measurements (Kotelnikova et al. 2017), as well as CELs (Gulati et al. 2015). Despite this, there remains significant work to be done if mathematicians are to have a reliable predicative capacity for MS. Limitations more broadly exist in all neuroimmunology, and while some mathematical modelling has been done on other neurological disorders (Elettreby et al. 2019; Sari et al. 2020; Santurtún et al. 2016; Menezes et al. 2016) there is still more to do.

Part of this review aims to motivate the mathematical community to fill-in-the-gaps that are currently missing in the literature. With this in mind, we provide below a list of challenges and open problems:

- ***MS disease as an epidemic:*** With the discovery of the correlation between Epstein Barr-Virus infection and MS (Attfield et al. 2022; Bjornevik et al. 2022; Bordon 2022; Sollid 2022; Yates 2022), mathematical modelling could help to understand how the likelihood of disease is correlated with EBV epidemiology.
- ***MS disease onset origin:*** Despite the knowledge of the link with EBV, the onset and origin of MS disease is largely debated, mathematical modelling of the hypothesises surrounding disease origin could shed some light on this problem.
- ***Early intervention leads to better prognosis:*** Potent ablation or suppression of immune cells early in the disease course is associated with reduced long-term disability in retrospective cohort studies, however, it remains unclear why this is (Attfield et al. 2022) and mathematical models of systemic immunology of MS could help.
- ***Heterogeneous lesion formation:*** Lesions are largely heterogeneous in shape and location, and it is unclear which dynamics drive the different spatial distributions and geometric configurations of lesions.
- ***Understanding and optimisation of disease treatment:*** Given the current lack of curative treatment, the long-term prognosis for MS patients is worsening disease progression. Can mathematics help us understand why the disease progresses despite intervention, and whether there are other targets in the MS disease network that might make more effective treatments?
- ***Spatial model of CNS inflammation:*** Modelling aimed at investigating the immune drivers of BBB breakdown and the importance of preserving its functionality in MS patients could give rise to therapeutic intervention points and increased understanding of disease progression. In addition, it would be valuable to develop models of cellular interactions in different neuroanatomical areas as we have evidence to suggest that immune responses in different regions of the CNS can be variable.
- ***Estrogen and Vitamin D:*** While it is well accepted that Vitamin D and estrogen play a major role in MS susceptibility, there are many questions that remain biologically surrounding their mechanisms of action.
- ***Hypothesis testing of MS immunology:*** testing of MS immunology: There is still much unknown about MS immunology. More recently there has been a conceptual shift in understanding the immune pathology of MS, away from a purely T-cell-mediated model to recognition that B cells have a key role in pathogenesis (Arneth 2019; Hauser and Cree 2020). This has not been investigated using mathematical modelling.

The above represents the open biological questions mathematicians could attempt to add insight to, and there are also opportunities to extend existing mathematical work. For example, approaching this disease from an epidemiological modelling perspective could shed some light on future disease impact on our community by building off current COVID-19 or other epidemiological models. In addition, within-host models of MS treatment would greatly benefit from a pharmacokinetic/pharmacodynamic modelling approach, which seems to be non-existent in the current literature. Lastly, the ABMs so far have focused on on-lattice cellular dynamics despite the white matter tracts of the brain being quite heterogeneous. Modelling cellular movement of MS specific agents in these tracks through off-lattice models may therefore give more realistic opportunities to understand the dynamics at play.

While there has been minimal attention from the mathematical community afforded to MS compared to other areas such as cancer, the work that has been done has provided a firm foundation through which future mathematicians can build upon to provide disease insight across the multiple scales (Fig. 3). The opportunities for mathematics to help improve the diagnosis, prognosis and treatment of this disease are vast, and mathematics could be the key to answering some of the major unknowns surrounding MS.

## Abbreviations

MS: Multiple sclerosis
CNS: Central nervous system
BBB: Blood brain barrier
RRMS: Relapsing remitting multiple sclerosis
PPMS: Primary progressive multiple sclerosis
SPMS: Secondary progression multiple sclerosis
EBV: Epstein Barr virus
MRI: Magnetic resonance imaging
CEL: Contrast enhancing lesion
EDSS: Expanded disability status scale
Tregs: Regulatory T cells
Teff: Effector T cells
DMT: Disease modifying therapy
IFN: Interferon
ODE: Ordinary differential equation
BST: Biochemical systems theory
RNFL: Retinal nerve fibre layer
PDE: Partial differential equation
SDE: Stochastic differential equation
ABM: Agent based model
PN: Petri net
PCA: Principal component analysis
ORC: Ollivier–Ricci curvature
